# Complementary and alternative health care in Israel

**DOI:** 10.1186/2045-4015-1-7

**Published:** 2012-02-20

**Authors:** Judith T Shuval, Emma Averbuch

**Affiliations:** 1 Rose Chair in the Sociology of Health, School of Public Health and Department of Sociology and Anthropology, Hebrew University of Jerusalem, Avizohar 8-671, Jerusalem 96267, Israel; 2 School of Public Health, Hebrew University of Jerusalem and Israel Ministry of Health, Ben Tabai 2, Jerusalem 91010, Israel

## Abstract

The paper explores the patterns of coexistence of alternative/complementary health care (CAM) and conventional medicine in Israel in the cultural, political, and social contexts of the society. The data are drawn from over ten years of sociological research on CAM in Israel, which included observation, survey research, and over one hundred in-depth interviews with a variety of CAM practitioners - many with bio-medical credentials - and with policy makers in the major medical institutions. The analysis considers the reasons for CAM use, number of practitioners, the frequency of CAM use and some of its correlates, and how CAM is regulated. The structure of the relationship between the conventional health care system and CAM is discussed in the public sector, which provides two-thirds of CAM services, and in the private sector, which provides about one-third. The history of the development of these structures and some of the dilemmas of their operation are discussed. A number of policy issues are considered against this background: regulation and licensing, CAM in primary care, reimbursement for CAM treatment, and the inclusion of CAM in education and training for the health professions.

## Introduction

Despite the vast achievements and dramatic successes of conventional bio-medicine, remarkable numbers of people in Western societies seek complementary or alternative health care (CAM). In most cases they do not abandon conventional medicine but turn to CAM as an additional mode of care [1-4].

The National Institutes of Health in the United States have provided scientific evidence for the effectiveness of some forms of alternative care, but many of the widely used methods have not passed rigorous tests for efficacy or have never been scientifically scrutinized http://nccam.nih.gov. The lack of scientific evidence hardly troubles users of alternative medicine: what counts for them is the fact that in many cases it works! - they have little interest in how or why. Failures are largely ignored while success is celebrated. It would seem that these social processes in the field of health care are not ephemeral but are likely to continue in one form or another. Indeed it seems fruitful to view CAM as an important component of the overall health care system. For this reason it is our hope that the Israel findings reported here will be useful to health policy planners in other countries as well.

The present paper draws on over ten years of research on CAM in Israel that included observation, survey research, and more than one hundred in-depth interviews with a variety of CAM practitioners - many with bio-medical credentials - and with policy makers in the bio-medical system. The overall goal of that research was to explore the multiplicity of empirical types of coexistence between alternative and bio-medicine that have emerged in Israel. These are seen in a variety of forms that reflect cultural, political, and social forces in the society. We sought to understand the dilemmas and quandaries that arise due to the perspectives of the principal actors who adhere to different beliefs and ideologies regarding the causes of health problems and appropriate therapeutic procedures [5-12].

In this paper we will draw on some of the findings of that research to consider the modes of delivery of CAM in Israel and its relation to the bio-medical care system. To set the scene, we begin with an overview of the Israeli health care system and define several key terms used in the paper. Next, we review the reasons for CAM growth internationally, and review the data on CAM utilization in Israel. This is followed by a discussion of the nature of CAM providers and CAM regulation in Israel. Then, the heart of the paper analyzes the structure of co-existence between bio-medicine and CAM in Israel, considering both the public and private sectors. The paper concludes with a discussion of a number of pending policy issues regarding CAM in Israel.

Our point of view is sociological. We do not seek to evaluate CAM, but rather to understand the implications of a phenomenon that plays an increasingly important - but hardly recognized - role in Israel's health care system.

## Setting the Scene [13-15]

In 2009 Israel had a population of 7.5 million, of whom 76% were Jews, 17% were Muslim Arabs, 3% Christians, 2% Druze, and 2% other. Immigration has played a central role in the society. In the decade 1990-2000, almost one million immigrants arrived, most from the former Soviet Union countries.

The 2009 GDP per capita income (with purchasing power parity (PPP)) was US$ PPP 29,404 [16]. Health care accounts for approximately 8% of the GDP.

In 2009 life expectancy at birth was 79.7 years for males and 83.5 for females. The infant mortality rate was 3.8 per 1000 live births, which has declined by 38% since 1996. The infant mortality rate for the Arab population has declined even more steeply, but remains double that of the Jewish population. This pattern reflects the influence of high rates of consanguineous marriage and lower socio-economic status among the Arabs.

In 2009 there were 3.4 physicians per 1,000 population. Of all licensed physicians, 41% were trained in Israeli medical schools while the remainder was trained abroad.

Since 1995, when the National Health Insurance (NHI) Law was formally enacted, all persons in Israel are covered by comprehensive health insurance, which includes curative and preventive care as well as hospitalization. Health care is available and accessible to the entire population. The Law provides access to a broad benefits package that includes physician services, hospitalization, medication, and other services. There is cost sharing for pharmaceuticals, visits to specialists, and certain diagnostic tests. The State, through the Ministry of Health, is responsible for supervising, licensing, and overall planning of health services.

Every citizen or permanent resident is free to choose among four competing, non-profit-making health plans, known in Israel as "sick funds": Clalit, Maccabi, Meuchedet, and Leumit. The respective market shares of the four sick funds in 2010 were: 53%, 24%, 14%, 9%. The sick funds provide their members with access to a benefits package that is specified within the NHI Law. The system is financed primarily through progressive taxation linked to income. The government distributes the NHI funds among the sick funds according to a capitation formula that is based primarily on the number of members within each plan and their age mix. The sick funds provide a broad network of easily accessible community-based clinics with salaried physicians and other health care personnel (for a comprehensive summary on health care in Israel, see [15]).

## Some definitions

We will use the term bio-medicine to refer to the commonly established form of Western medicine taught in most medical schools, which exercises a dominant, often exclusive, monopoly over legitimate medical care in many societies.

Physicians generally prefer to use the term "complementary" to refer to unconventional modes of health care. This stance reflects a medicocentric view, which implies greater validity and centrality to bio-medical procedures and a lesser status to unconventional practices that "complement" them. The term "alternative" is viewed by many in the medical establishment as offensive and challenging to their exclusive hegemony.

In order to avoid the pitfalls associated with both of these concepts, in this paper we will use the term CAM (complementary and alternative medicine) to refer to the combined array of non-conventional health practices commonly in use in Western societies.

These have also been referred to as holistic, natural, unorthodox, fringe, and unconventional ([17], p. 480). The National Center of Complementary and Alternative Medicine (NCCAM) defines CAM as "a broad domain of healing resources that encompasses all health systems, modalities, and practices and their accompanying theories and beliefs, other than those intrinsic to the politically dominant health system of a particular society or culture in a given historical period" ([18], p. 50).

While aware of the heterogeneity of CAM practices and the broad array of epistemological grounds on which they are based, we will follow Montgomery and Keshet's precedent and, for purposes of the present paper, consider them as a whole [2,19].

Despite the differences among its many modes, a number of common characteristics of CAM have been noted and may be viewed as paradigmatic. Most critical is a holistic perspective, which recognizes that people are simultaneously biological and social creatures and that biology and culture interact as equal partners in defining ''who and what we are" ([19,20], p. 154). Illness is viewed as an imbalance in energy forces and a failure of the body's restorative powers. Germs in themselves are not viewed as the cause of disease. Thus there is an emphasis on assisting patients to heal themselves. In addition, CAM emphasizes individuality, interpersonal interaction of practitioners with patients, subjectivity of experience, feeling, energy balance, and prevention. The logic of treatment is seen in a focus on the patient whose body or mind (or both) will then initiate the healing process [1,3,21-24].

The principal forms of CAM practiced in Israel include homeopathy, Chinese medicine, acupuncture, herbal medicine, reflexology, Reiki, shiatsu, chiropractic, biofeedback, Ayervedic Medicine, naturopathy, massage techniques, Bach flower remedies, Feldenkreis, anthroposophy, Twina, osteopathy, Paula (a system of orifice muscle exercises developed in Israel), and others. While this list is not comprehensive, it includes the forms of CAM practice that have generally been incorporated into the clinics of the public medical care system in Israel and in many private clinics. Folk and traditional forms of health care, which may also be viewed as a form of CAM, have not been incorporated into the medical care system as above and therefore are not discussed in the present paper.

## Reasons for the growth of CAM

There are a large number of reasons suggested in the literature for the growth of CAM since the 1980s. Only some of them have been examined empirically. We will refer to those most frequently cited [1,4,25-28].

Increased use of CAM has been explained by objections of consumers to the invasiveness and excessive use of the technology of bio-medicine. Consumers are increasingly aware of iatrogenic effects of modern medicine and prefer to ingest fewer drugs. Many object to the traditional dominance of doctors often seen in the physician-patient relationship. In a period of hyper-differentiation in biomedicine, when it is practiced in large organizations where there is minimal attention to the individual and to her or his social and psychological needs, CAM offers a non-invasive, holistic alternative that is increasingly attractive to many, in particular to the better educated more affluent segments of the population. There is more awareness among consumers of the relationship of lifestyle to morbidity, especially when bio-medicine is unable to provide relief or cure. It has also been noted that in the post-modern period, with on-going globalization, there has been an overall decline in faith in the ability of science and technology to solve health problems. This is seen in the lesser acceptance of traditional authorities such as physicians and a seeking by individuals of increased control over their life and health. Globalization has been accompanied by increased migration of populations and the transmission of therapies and medical theories among different societies. These factors have combined in Israel, as in other nations, with demographic changes seen in an increased prevalence of chronic health problems that are less responsive to the methods of biomedicine [29-31].

## Use of CAM

Use of CAM in Israel began to grow in the 1980s [32] and has continued to increase since then. This has not reduced the utilization rates of the bio-medical care system but presents challenges to its monopolistic authority and hegemony.

Data from a survey in Israel by BDI-COFACE in 2008 indicate 1,750,000 CAM visits a year or 145,833 a month (http://www.bdi.co.il/EngDefault.aspx, 2008). The most recent data concerning patterns of use of CAM in Israel are drawn from a study based on a sample (N = 1635) of the Israeli Jewish urban population aged 22-75 in 2007 [33,34].

In 2007, 12% of the population reported using CAM at least once during the previous year. More than a third of CAM users reported consultations with more than one type of CAM practitioner. A study by the Brookdale Institute showed similar findings in 2009 [35].

Although the use of CAM has increased in Israel in recent years, Israel ranks among relatively "light" users in comparison to other Western countries. Use of CAM was most prevalent among women, the educated, economically well-established, and married persons.

More than half of CAM users reported using it to treat a specific health problem. The most frequently mentioned were back pain, problems with joints and limbs, and general health. The most common reasons given for seeking CAM therapies focused on the perceived negative aspects of bio-medicine. General disappointment and dissatisfaction with conventional treatment, unwillingness to take many medicines or to use invasive care, and the failure of bio-medical efforts to alleviate a problem are common reasons for use of CAM. At the same time, the number of people who reported using CAM following positive personal experience or recommendations by others was also high.

The most frequently used therapy was acupuncture (37%), about a third were treated with reflexology, 29% used homeopathy and 26% reported using massage, less than a fifth used chiropractic therapy (16%). Most of the referrals to CAM practitioners were initiated by the individuals themselves, a fifth followed recommendations by friends and family members.

The percentage of users referred to CAM by a physician increased significantly from 6% in 1993, to 10% in 2000 and to 17% in 2007. This increase suggests a growing acknowledgement and acceptance of CAM by the medical establishment [34].

Among users of CAM, about 80% reported that the therapy relieved their suffering. Two-thirds of the users reported high general satisfaction with the CAM provider they used, and very high satisfaction was reported in all aspects of the practice: the provider's attitude, the time dedicated by them, their availability, the information provided by them, and the quality of the therapy. More than half said they would recommend the use of CAM to others.

Thirty percent of CAM users received bio-medical therapy for the same problem while 70% used CAM instead of conventional medicine. More than half of the CAM users did not disclose their use of CAM therapy to their family doctor. The reasons for nondisclosure varied. The common ones were, "It was none of the doctor's business and I do not care what his/her opinion is", and "The doctor has no knowledge about CAM, never asks about it or is against it". When users were asked what they think their doctor's attitude to CAM therapies is, most of them said that they think their doctor does not oppose the use of CAM. No data are available to indicate the implications of this non-disclosure on patients' health.

The findings from this study carried out in Israel in 2007 do not differ substantially from the findings of similar studies concerning CAM use in other Western countries [33].

## CAM Providers

In 2011 there were an estimated 20,000 CAM practitioners working full time and part time in Israel. Of these only 2,800 are members of professional organizations representing a wide variety of CAM specialties (personal communication from Mr. Ofir Sela, chairman, Association of Complementary Health Care Organizations, (HaLishka Lemikzto'ot Briut Maslimim), May 2011).

There were approximately 60 programs for teaching CAM in Israel in the framework of courses lasting between three months to four years. The courses vary widely in the quality of training they provide. There is no supervision or control regarding content. When a well known college sought to incorporate the teaching of CAM into its undergraduate program in 2009, its proposal was rejected by the Council on Higher Education.

The four medical schools vary in their attitudes to CAM. Some include short elective courses *about *CAM but none include such courses in the compulsory curriculum. At the same time, Israeli medical students show a high level of interest in CAM: 79% of medical students in their last year of study expressed an interest in learning about CAM during their course of study; 65% stated that they would be interested in applying CAM techniques to treat patients [36]. This finding is strikingly similar to an earlier finding by Schachter et al. [37] in their study of the attitudes of Israeli family physicians toward non-conventional medicine. They found that 87% wanted to know more about CAM therapies and 78% believed that CAM should be taught in medical school. It seems safe to assume that these percentages have increased in recent years.

In 2002, at the initiative of a small group of members of the Israel Medical Association, the Israel Society for Integrative Medicine was founded within the framework of the IMA. It includes members of the Association who practice both bio-medicine and CAM and seek to integrate them with each other. By 2009 it had 150 members. The very existence of the Israel Society for Integrative Medicine is an important indication of the growing recognition by the medical establishment of the legitimacy of CAM practice by qualified physicians [12].

## Regulation of CAM

Israel has never established any formal jurisdictional regulation or control of CAM. There is no licensing procedure, little teaching of CAM in the medical schools, and no regulation of the many courses and schools which train CAM practitioners. (See [12] Appendix B, for details regarding the legal status of CAM in Israel.)

The only relevant jurisdictional control mechanism is the Doctors' Ordinance [38], which provides that only persons holding a physician's degree and license may practice medicine. The Eilon committee, set up by the Ministry of Health in 1988 to examine the status of CAM in Israel, recommended that the above law be changed to make explicit the individual's basic right to select the health practitioner of his choice. At the same time it sought to establish appropriate licensing of CAM practitioners in order to guard against incorrect diagnosis or treatment that could cause health risks or harm to patients. It proposed that only licensed physicians be permitted to use the title "doctor" and that CAM practitioners adopt the ethical code of the medical profession. Finally, it encouraged research on the effectiveness of CAM [39].

None of these recommendations presented in 1991 were accepted. Indeed the Israel Medical Association issued a number of public declarations during the following decade seeking to assert the unquestioned hegemony of bio-medicine. In 1997 the IMA stated in one of its widely circulated publications [40] that "There is only one form of medicine which deals with care of human beings and only physicians are permitted to practice it...". However, in an apparent allusion to CAM, the same statement notes that "... within the context of professional practice, physicians may select a variety of methods" [41,42].

The Ministry of Health, despite prolonged deliberations, has never been able to reach a formulation regarding regulation that was acceptable to the relevant interested parties, e.g., Israel Medical Association, CAM professional associations, and the sick funds. The result has been a reinforcement of the monopoly of bio-medicine by denying independent access to the provision of health care by non-physicians.

In 2003 an additional committee was set up by the Ministry of Health to formulate a licensing procedure. During its deliberations, it considered a proposal for regulation by the state and academization of CAM studies. Incorporating CAM into the university programs was strongly opposed by some of the CAM practitioners who run their own schools for training practitioners. While this proposal was under consideration, one of the elite colleges submitted a request to the Council for Higher Education for the establishment of an undergraduate program of CAM studies. In 2009 the Council for Higher Education rejected this request. To date the committee has not submitted a final report.

## The Structure of Co-existence - Bio-medicine and CAM

We will first discuss the large public sector CAM services and then describe the smaller private sector.

### 1. The Public Sector

Sixty-five percent of CAM services in Israel are provided in the public sector of the health care system: by three of the sick funds and about one-third of the hospitals (http://www.bdi.co.il/EngDefault.aspx, 2008). The number of clinics run by each of the sick funds in 2010 reflects their relative size: Clalit - 50 clinics, Meuchedet - 16 clinics, Maccabi - 16 clinics; they are located in the major urban centers. Kupat Holim Leumit does not run a separate network of CAM clinics but includes CAM practitioners in 86 of its regular community clinics.^2 ^Outpatient CAM clinics are run by eleven of the public hospitals. In addition, a small number of primary care physicians provide integrative care within the framework of the regular sick fund clinics.

We will discuss each of these forms of delivery in turn.

#### a. CAM community clinics

The first CAM-dedicated clinic, established in 1991 at Assaf Harofe Hospital, established a structural pattern that was subsequently adopted by all of the community CAM clinics run by the sick funds as well as the out-patient clinics associated with the hospitals. Close examination of the mode of operation of these clinics in the 1990s and again in 2010 shows that the initial pattern has persisted in its original form for close to two decades ([12], Chapter 5).

The structure devised for the CAM clinics reflected three goals of the bio-medical initiators: to gain as much income as possible from patients' fees; to maintain the hegemony of bio-medicine; and to attract clients by offering a broad array of CAM services.

The CAM clinics require a fee for service. This contrasts with the regular sick fund clinics in Israel where medical care is dispensed with no fee for service or with a small symbolic charge for specialists. These fees are controlled and persons carrying supplementary health care insurance (74% of the population in 2007) pay about half of the fee [43]. In the private sector fees are 2-3 times higher. Like all payments for health care, the fees introduce an element of inequality by making CAM less accessible to lower-income segments of the population.

The structure of the clinics may be viewed as a form of co-optation that attained all of the above goals. It was constrained by the jurisdictional regulations of the Doctors' Ordinance, which provides that only a licensed physician may practice medicine ([38], Appendix B). Since the Ordinance also permits qualified persons working under the supervision of a licensed physician to provide health care, this authority is sufficient to legitimize the work of CAM practitioners in the clinics as long as they are supervised by a physician [41,42,44].

Re-asserting its traditional hegemony, the bio-medical institutions entitled the newly established CAM clinics "complementary". The notion of "alternative" was deliberately rejected. This labeling strategy - one of the processes termed "boundary work" by Gieryn [45] - highlights the dominance of bio-medicine and makes an unequivocal statement regarding the secondary role of CAM. The Hebrew term "refua mashlima" makes clear that CAM adds to or completes the work of the bio-medical component.

The modus operandi of the clinics is as follows: The director of each clinic is a bio-medical physician who is also qualified in one or more fields of CAM practice. The other practitioners employed in the clinics include CAM specialists in many fields: acupuncture, homeopathy, chiropractic, reflexology, Feldenkreis, Reiki, naturopathy, touch and movement modalities, herbal medicine, biofeedback, Alexander, aromatherapy, alternative nutrition, Paula, and others. Only a minority are also licensed in a bio-medical field.

A bio-medical screening process establishes an unmistakable boundary defining the domains of bio-medicine and CAM: at the time of their first arrival, all clinic patients undergo a preliminary screening routine based on their bio-medical records. If these are incomplete, the patient is requested to complete them before she/he can be accepted for CAM treatment. The senior physician - generally the director of the CAM clinic - serves as a diagnostician and administrator but does not practice as a bio-medical clinician in these settings although he/she may practice in a CAM specialty.

The hegemony of bio-medicine defines a hierarchical relationship with CAM. This is seen in the salaries and employment status of the CAM practitioners, which is inferior to that of the bio-medical personnel [8].

At the same time, it has been noted that its acceptance within the bio-medical organizational structure endows CAM with a significant measure of legitimacy regarding health care [46]. Despite the ambiguous nature of its jurisdictional status, its sponsorship and inclusion within the broad boundaries of the prestigious bio-medical family of health care institutions bring it close to mainstream health care in Israel. What is more, patients gain confidence in CAM from its bio-medical sponsor [12].

Our research has shown that the over-all, bio-medical control of the CAM clinics leaves a large measure of autonomy to the CAM staff. While the formal control mechanisms remain, they play only a minor role in the context of everyday clinical practice in which CAM is primary. From the CAM practitioner's viewpoint, the bio-medical control function is felt sporadically - before the patient reaches the stage of CAM treatment and again at the end of a series of CAM treatments for an evaluation session. What is important is the fact that CAM practice is segregated from bio-medicine and there is little integration of skills representing the two spheres. Nor is there evidence to suggest that the CAM practitioners feel a need for greater bio-medical contacts. We did not find any meaningful dialogue regarding theoretical or epistemological issues between bio-medical and CAM practitioners ([12], Chapter 5). In terms of the model suggested by Boon et al. [47], this form of practice may be seen as "parallel" rather than integrative medical practice.

In such a work environment there exists an opportunity for interaction and consultation among CAM practitioners with different specialties. However, our research found this to be a relatively rare phenomenon. Like most bio-medical specialists, the CAM practitioners tend to remain within the confines of their own specialty - unless specifically approached for a professional opinion. This is a form of isomorphism in which CAM practitioners re-enact the professional behavior of their bio-medical counterparts [48].

#### b. Hospitals - CAM practitioners in bedside care

Our early research in 2000 found CAM practitioners to be thinly spread in a number of hospitals in numerous departments: orthopedics, oncology, pediatrics, internal medicine, obstetrics, premature infants, pediatrics, gastroenterology, neurology, and pain clinics. The latter were among the first to accept CAM practitioners because of the widespread awareness among bio-medical practitioners that they do not have effective means of dealing with pain. At that time, no one department contained a critical mass of CAM practitioners such as to be visible to the public or the staff. CAM practitioners working inside hospitals encountered numerous barriers ([12], Chapter 5; 13), which made it clear that their presence was met with considerable reservation.

At that time there was little contact between hospital-based outpatient CAM clinics and in-patient care. By the end of the decade we found that the CAM presence inside Israeli hospitals had increased, had become more visible and in a variety of forms. For the large part, the changes were spearheaded by individual initiatives undertaken by energetic physicians who were imbued with a keen desire to establish integrative medical care. Many are inspired by examples from the U.S. on which they seek to model their services, especially in the field of oncology (Memorial Sloan Kettering Cancer Center, Dana Farber Cancer Institute, M.D. Anderson Cancer Center).

The changes were brought about by individuals rather than by formal policy decisions of hospitals or the Ministry of Health. For this reason the changes initiated differ from hospital to hospital and depend to a large extent on the interests or specialization of the local bio-medical initiator.

The most developed CAM programs in the hospitals at the end of the decade are geared to accompany bio-medical oncological treatment. Such programs have been established inside several hospital departments or near them. They offer patients a wide variety of therapies to assist in alleviating the side effects of radiological treatments and chemotherapy, reduce tension, lessen pain, and strengthen coping strategies. The CAM techniques utilized include acupuncture, herbal remedies, naturopathy, shiatsu, reflexology, touch therapies, hypnosis, bio feedback, meditation, yoga, tai chi, chi gong, and others. Some programs offer workshops in yoga, tai chi, and psychological support services to patients and to their families. Information and guidance are provided on nutritive supplements and herbal products.

Within this context, bio-medical modes prevail. Patients are assured of bio-medical supervision and consultation services in the CAM setting as well as coordination with their primary bio-medical oncological therapist in the hospital. Informed consent is a pre-condition for treatment by CAM. Specialization is seen in the fact that some hospitals treat only adults while children are treated in a setting that specializes in pediatric oncology (http://www.rmc.org.il).

Most of these services require payment of a fee with reduced charges offered to members of specific sick funds that have contracted with the CAM clinic. Members of other sick funds pay a full fee. As noted, in all cases the financial outlay for a series of treatments is far from negligible.

Some activities inside hospitals are supported by grants for research that seeks to provide systematic evidence for the effectiveness of CAM treatment. All of the CAM clinics - which are directed by integrative physicians - make a point of highlighting their clinical research and teaching programs, which provide legitimization for their presence in a bio-medical setting. This strategy has been used in expanding CAM into the hospital system. Careful documentation of procedures and recording of patients' reactions to various CAM modalities accompany some of the treatment processes. In a few hospitals the research procedure legitimizes entering the data into the patient's medical record - an innovative process that has long been advocated by integrative physicians. Such records have a long-range impact, imparting increased legitimacy to CAM procedures.

Like in other countries, the establishment of CAM services inside hospitals is often dependent on a "motivated champion" - an individual or family who takes the initiative to recruit support and funding for CAM services. Such champions are generally lay persons who have themselves benefited directly or through family members from CAM treatment. They are active in mobilizing support for CAM at a variety of levels in the bio-medical hierarchy. The initial arrangements for organizing these services are informal and based on particularistic relations with biomedical figures who are unable - or unwilling - to seek support for CAM inside the official administrative structure of the hospitals [49].

In recent years, the labeling of CAM in hospitals has begun to change. As noted, in the past CAM was labeled complementary to medicine. Recently some of the hospitals have expanded the formal title of the CAM services to include the term integrative. This change carries important symbolic implications. Rather than viewing CAM as a secondary complement to bio-medicine, the term "integrative" highlights the partnership of bio- and alternative medicine.

While oncology is the most prominent area into which CAM has expanded, it has been developed in other hospital departments as well. Among these are neurology, pain clinics, dermatology, gastroenterology, and orthopedics. In addition, a gynecology department offers CAM treatment to menopausal women. A pediatric CAM clinic at one of the hospitals offers care for sleep disturbances, digestive problems, respiratory difficulties, headaches, anxiety and depression, and behavior problems. Pregnant women are offered CAM assistance by midwives before and during birth at a number of hospitals. A preventive program in a cardiology department offers CAM treatment in addition to its bio-medical program: to reduce high blood pressure, control unstabilized diabetes, prevent obesity, and stop smoking.

In the past, departments of surgery and especially operating rooms have been strictly off-bounds to CAM in all Israeli hospitals. In 2010 an experimental program was activated in at least one hospital utilizing CAM in pre- and post-surgical settings. Techniques such as hypnosis, reflexology, acupuncture, bio-feedback, guided imagery, and touch techniques have been used to control anxiety and tension, ease pain, reduce nausea, control post-operative complications, and speed recovery. The experiment is defined as a research project: thus all of the CAM procedures are meticulously monitored including patients' response to treatments. These are recorded in the patient's bio-medical clinical record. While the operating theatre itself remains closed to CAM, the boundaries of its surrounding territory in which critical pre- and post-surgical procedures occur, have been re-contoured to admit CAM practitioners.

We may conclude that the presence and activity of CAM in Israeli hospitals has increased over the past ten years - principally as a result of energetic efforts by individual directors of outpatient CAM clinics who embraced the mission of promoting integrative medicine inside the hospitals.

The introduction of palliative and rehabilitative care of cancer patients served as an effective spring-board for the introduction of CAM into the heart of hospital practice because of its focus on care rather than cure [50]. Creative use of evidence-based research regarding CAM has promoted the respectability and acceptability of CAM in settings where it was previously rejected. It has also induced important changes in the protocol of hospital care, e.g., the inclusion of the record of CAM treatment in the patient's file.

In this sphere, as in others, isomorphism plays a role: the more CAM looks like and *feels *like bio-medicine, the greater its acceptability in the hospital system. The promoters of integrative medicine are essentially medicalizers of CAM - but at the same time "CAMifiers" of bio-medicine. The former is for the moment the more powerful force - but the latter is far from negligible and is likely to increase in the future.

#### c. Integrative Care in Sick Fund Clinics

In 2009, there were over 5,000 physicians practicing in the regular primary care clinics run by the four sick funds. These are distributed in all parts of the country and provide universal medical care under the provisions of the National Health Law of 1995 [15]. As noted, CAM is not included in the list of entitlements under the Law.

Among these physicians is a small sprinkling of primary care doctors who are also qualified in one or more CAM fields and who seek to integrate these skills into their practice. Most of them are family practitioners, internists, or pediatricians.

These initiatives in integration are individually motivated and do not reflect a policy stance on the part of any organized health care institution. None of the three sick funds that employ these physicians has addressed the many issues involved in this innovative mode of practice. Insofar as the sick funds are concerned, CAM is officially provided in the special network of clinics dedicated to CAM which they have established.

The CAM physicians who work in the regular sick fund clinics are unbothered by epistemological or cognitive conflicts and generally believe in "one medicine". Many see themselves as pioneers and are imbued with zealous enthusiasm to utilize the best of their bio-medicine and CAM skills for the benefit of their patients. They seek fully integrated medicine in which bio-medical care and CAM are practiced jointly.

The integrative physicians are engaged in a process of CAMification of primary care, i.e., introducing elements of CAM when they believe they will improve the quality of clinical practice. Like CAM midwives ([13], Chapter 9), they identify strongly with their bio-medical profession while at the same time seeking to introduce CAM into clinical encounters when they believe it can be useful.

The research shows that the organizational boundary drawn by the sick funds presents a major obstacle to the implementation of this type of integrative practice. Since CAM is not included in the basic list of entitlements provided by law, it cannot be provided in the context of the sick fund clinics without a fee for service. The salaried primary care doctors are not permitted to take fees during their working hours. As mentioned earlier, from the point of view of the sick funds, the demand for CAM is formally dealt with by the network of CAM clinics and patients should be referred to them. By adopting this stance the sick funds in effect support the "parallel" style of integration practiced in the CAM clinics.

These constraints cause considerable discomfort to the integrative physicians who are constrained in the use of their CAM skills. Many are critical of the quality of care offered in the sick funds' CAM clinics and of the separatist mode of practice there which differentiates bio-medicine from CAM so sharply. At the same time, it is awkward for them to refer patients to their own private clinics.

Despite their desire to integrate CAM into the public primary care system, some decide that part-time private practice provides a preferable solution. Many have opened private clinics in which they integrate bio-medicine and CAM, giving primacy to whichever type of treatment seems optimal for individual patients. In these settings CAMification is more easily achieved.

An additional option is to introduce CAM discreetly into the public primary care clinic - by offering patients CAM advice and therapy informally, as best they can. Most amenable to such discreet entry are recommendations regarding alternative diets, relaxation techniques, and use of herbal or homeopathic products that can be purchased over the counter. Time- and space-consuming procedures - such as acupuncture or reflexology - are more difficult to introduce.

Research shows that CAM midwives are in a similar position: working in the delivery rooms of hospitals, they introduce CAM procedures discreetly, with the consent of the birthing woman. The environment of the delivery rooms is strongly bio-medical, but the CAM midwives resist the medicalization of childbirth. They seek to reduce the use of epidurals while introducing massage and other touch techniques to control the pain of delivery. Their work is subject to bio-medical scrutiny by other midwives, but they are supported inside the hospital by those obstetricians who promote natural childbirth. This approach of the midwives provides a further example of the CAMification process ([10-12], Chapter 11).

### 2. The Private Sector

The percentage of private participation in the global expenditure on health has been increasing in recent years in Israel. In 2010 it reached 43%, which is higher than in most developed OECD countries (http://www.oecd.org). This is a source of serious concern in a society committed to an egalitarian policy in health care. The fact that 35% of CAM is provided by the private sector contributes to inequality and is part of the broader trend seen in the on-going reduction in public support of health and welfare programs.

Part of the private sector delivering CAM services is linked into the public sector by contracts and agreements with the sick funds, which provide reduced fees to patients carrying supplementary health insurance.

Many of the CAM practitioners in the private sector also work in the public sector. The lucrative setting of private practice is seductive in a society that is structured around an open market economy. Working privately on a part-time basis, practitioners are able to augment their incomes considerably.

Private practice offers practitioners a less constrained clinical ambience: they have more time to select their diagnostic and treatment options as they see fit. For practitioners who are bio-medically trained, the balance of bio-medicine and CAM is not constricted and can be freely adjusted by the practitioner to a patient's needs. In this context CAMification is an open option.

CAM nurses provide an example in which a spatial-temporal boundary provides for complete separation between their bio-medical and CAM practice. These nurses practiced bio-medicine in public hospital settings and moved to a private clinic elsewhere to practice CAM. In each of these differentiated settings the nurses work in an environment that is minimally invaded by values or controls that challenge the treatment modes they choose to utilize. They do not worry about significant others scrutinizing their clinical work. At the same time, we have noted an interesting asymmetry in this type of differentiated boundary work. In the context of their private CAM clinic the nurses are entirely comfortable in importing bio-medical skills over the territorial boundary defining their CAM environment. Thus they view it as a sine qua non to screen patients by the use of bio-medical tests and records before starting CAM treatment.

However, in the context of their hospital work, the nurses adhere strictly to bio-medical procedures. Even when they believe that a CAM technique could relieve a patient's pain, they are reluctant to use it - lest one of the other bio-medical members of the staff in the department observe such deviant behavior. Sometimes they use CAM discreetly - while checking over their shoulder for a critical presence ([12], Chapter 8).

## Some Policy Issues

### Regulation

The public as a whole and patients in particular are entitled to a safe system of CAM care. It is the responsibility of the state to protect the public by ensuring that CAM is practiced in accordance with professional, ethical, and practice standards [51].

A major outstanding issue in Israel - as in many other countries - is the establishment of a system of licensing that makes explicit and enforceable the standards of practice required for the different forms of CAM. The absence of formal regulatory legislation to control CAM practice, education, and licensing is not unique to Israel [52]. However, there are a number of countries that have begun the process of regularization and Israel could learn from their experience [53-58].

In Israel, although a number of public committees have presented recommendations regarding this issue, none have been accepted. The result has been that the Doctors' Ordinance of 1976 remains the sole jurisdictional mechanism from which CAM practice may attain legitimacy. This Ordinance provides that only licensed physicians may engage in medical care but physicians may supervise the clinical work of unlicensed health care practitioners. It is the latter provision that provides legitimacy for CAM practice by non-physicians in the CAM clinics.

The result has been the establishment in Israel of a highly medicalized network of CAM clinics within the system of publicly sponsored medical care. Responsibility for quality control is placed solely in the hands of physicians, even though it cannot be assumed that training for medicine in itself provides the knowledge, skills, and judgment needed for this task.

Licensing for non-medical CAM practitioners is a complex problem that needs to be addressed creatively taking into consideration the considerable differences among the various CAM specialties and their specific needs. Israel can learn from the experience in Europe and in the United States where guidelines have been developed for the appropriate practice of CAM [51,56,59].

### Primary care

The structure of the CAM clinics in Israel does not encourage - and may discourage - use of CAM for primary care. CAM techniques are time consuming and can hardly be fitted into the prevailing norm of the sick fund clinics, which is six patients per hour. The organizational barriers to the incorporation of CAM into primary care are related to the difficulty of combining services covered by supplementary health insurance or requiring a full fee for service into the universal, free primary care clinics. This is discussed above.

These problems help explain the relatively "light" use of CAM in Israel in comparison to other Western countries where it is offered as a "direct form of primary health care" [60]. In England half of the general practices offer their patients access to CAM services [61]. In Germany 60% of the family doctors provide CAM in the context of their practice. This is the highest rate reported in the literature: the percentages range from 13% to 38% in other Western countries [62,63].

Indeed, the formal structure of the CAM clinics in Israel appears to be geared to patients who have not been able to obtain help in their regular bio-medical facility, so that in many cases CAM is viewed as a "last resort". In addition, the fee for service is a deterrent in a country where accessible primary care is available at no direct cost on a universal basis. Furthermore, the screening process imposes a structural delay in access to immediate health care: it requires an appointment with the senior physician and only after that has been cleared can a patient make an appointment with a CAM practitioner. The structural separation from bio-medical care also serves to discourage use of CAM for primary care.

An interesting experimental primary-care clinic that provides integrated CAM and bio-medical care in Sweden is described by Sundberg et al. [64]. The clinic included one general practitioner and eight CAM practitioners in different types of practice, all of whom worked on a part time basis.This experimental clinic, which is structurally similar to the Israeli CAM clinics, is relevant to the Israeli experience because it addresses issues of hierarchy and collaboration among bio-medical and CAM practitioners.

Hollenberg [65] highlights the difficulties inherent in integrative practice in an analysis of the relations between physicians and CAM practitioners in such settings. He points to the complexities of working together since bio-medical doctors often continue to perpetuate patterns of medical dominance by maintaining control of overall patient care and using bio-medical language as the primary form of communication. Research indicates that physician-managed CAM clinics often frustrate and stifle CAM practitioners [66].

Unlike the senior physician in the CAM clinics in Israel (rofe memayen), the "gatekeeper" in the Swedish primary care clinic is a bio-medical-CAM practitioner who is responsible for the full *clinical *management of the patient including recommendations for both bio-medical and CAM treatment. These recommendations are considered by a "consensus case conference" with the full provider team. The authors emphasize that a deliberate effort is made to avoid medical dominance in an effort to attain "interdisciplinary, non-hierarchical decision making involving a mix of conventional and complementary medical solutions in individual case management" [64].

The interesting difference between the Swedish experiment and the Israeli CAM clinics focuses on the role of the "gatekeeper": in Israel that role is supervisory and administrative with only a minimum of clinical functions; in Sweden it is supervisory in an egalitarian mode but also clinical [64]. In Israel there is no bio-medical care offered at the CAM clinics; CAM is provided in an insulated context. Both bio-medicine and CAM are present but they function separately with little on-going contact between them. Israeli policy makers might well learn from the Swedish experiment

### Reimbursement for CAM treatment

In an overview of systems of reimbursement for CAM treatment carried out in 23 countries and within the European Union, it was found that all countries have some form of social insurance and in some there are private systems of insurance as well [67]. The coverage of CAM varies. It can be complete or restricted by number or type of treatment; sometimes a matching payment is required. There are countries in which there is no reimbursement for CAM by public or private insurance; in other countries CAM is covered only by public or only by private insurance systems; and, finally, there are countries in which CAM is reimbursed by both public and private insurance systems.

In Israel a combination of the second and third system has been devised: the sick funds, which provide a universal set of health care entitlements, do not include coverage of CAM but sell supplementary insurance that covers partial coverage of CAM. Three-quarters of the population have purchased supplementary insurance from the sick funds or from other insurance bodies and are therefore partially covered for the cost of CAM treatment. Nevertheless, the costs to the consumer of CAM care contribute to inequality in health. Other countries with public/private systems of insurance are France, Italy, Germany, and Switzerland. In those countries only selected forms of CAM are covered, while in Israel all of the types of CAM offered in the clinic are included [67]. The pros and cons of the various approaches to financing CAM should be analyzed, along with their implications for Israeli health policy.

### CAM in Bio-Medical Education

CAM is offered sporadically in medical schools in Israel as an elective course; it is not a regular part of the curriculum in schools of nursing, of occupational therapy, or physical therapy. Practitioners in these schools and in other bio-medical training institutions need to be exposed to the basics of CAM therapies. The object is not to train them as CAM practitioners, but to provide a basis for understanding the options available to their patients for referrals. Furthermore, they need to understand the CAM experiences of those of their patients who have already undertaken these types of treatments. Greater understanding of CAM by bio-medical practitioners is likely to increase their open-mindedness so that patients will report to them more freely on their experiences with CAM treatment. Lack of knowledge or awareness of a patient's de facto use of CAM can be an impediment to appropriate bio-medical care. Research has shown that in the future CAM treatments may be included in patients' medical records and will therefore require more knowledge and understanding of its implications by bio-medical practitioners [13,68].

Curriculum committees, which determine course content and requirements in the medical schools and in the schools for other health care professionals, should explore the inclusion of CAM in their respective required curriculum.

## Conclusions

CAM is here to stay and its use is likely to increase in Israel in future years. Indeed the trend over the past decade points in that direction. Israel - at present a relatively low user - is likely to come closer to the levels seen in other Western countries for many of the same reasons seen there. It is of interest to note that research in Holland has pointed to a negative relationship between confidence in bio-medicine and use of CAM [27]. With the growing transparency of bio-medical procedures and widespread media coverage, it is possible that public confidence in bio-medicine may further decline, thus augmenting the likelihood of increased use of CAM in future years.

This means that the policy issues noted will become more urgent. Health policy planners can no longer put off, avoid, or ignore CAM's role as part of the overall system of health care. For this reason its control and regulation are essential. Furthermore, the Israel findings may also be useful to health policy planners in other countries in which CAM provides increasing segments of health care.

## Competing interests

The authors declare that they have no competing interests.

## Authors' contributions

The present paper was planned jointly by JTS and EA and drafted first by JTS. EA was consulted during this process, added a number of important additional ideas and checked the final version for accuracy and coverage. Both authors read and approved the final manuscript.

## Authors' information

Judith T. Shuval received her Ph.D. in sociology from Harvard University. She is Professor Emerita at the Hebrew University of Jerusalem, where she holds the Louis and Pearl Rose Chair in the Sociology of Health. She has received the Israel Prize for the Social Sciences.

Emma Averbuch received her PhD in Sociology from the Hebrew University of Jerusalem in 2010. She coordinates activities regarding coping with inequality in the Israel Ministry of Health and teaches sociology of health at the Braun School of Public Health, Hebrew University-Hadassah Medical School in Jerusalem.

## Endnotes

^1^Data on use of CAM by the total Arab population is not available, but findings of surveys carried out in specific areas are discussed in ([23], Chapter 12).

^2^See: http://www.clalitmashlima.co.il, http://www.maccabitivi.co.il/153-he/Maccabi_natural.aspx, https://www.meuhedet.co.il/meuhedet/downloads/extra.pdf, http://www.leumit.co.il/natural.asp?pgId=69&catId=497
